# Facilitators and barriers to condom use among Filipinos: A systematic review of literature

**DOI:** 10.34172/hpp.2020.49

**Published:** 2020-11-07

**Authors:** Ryan Q. De Torres

**Affiliations:** College of Nursing, University of the Philippines Manila, Manila, Philippines

**Keywords:** Condoms, Health education, Health promotion, Sexual health, Systematic review

## Abstract

**Background:** Between 2010 and 2018, the Philippines had a 203% increase in new human immunodeficiency virus (HIV) infections. The use of condoms is an effective and practical means to prevent HIV transmission. The purpose of this study was to identify facilitators and barriers to condom use among Filipinos guided by the Ecological Model of Health Promotion.

**Methods:** A systematic review of literature using electronic databases was performed using the following keywords: "condom," "Filipinos," and "Philippines." To be included in this review, papers should be (1) research studies, (2) studies that examined condom use, and (3) studies that sampled Filipinos residing in the Philippines. The final sample comprised of 27 articles.

**Results:** Multiple and interrelated factors at the individual and social environment levels influence condom use among different groups of Filipinos. Majority of these factors originated at the intrapersonal level. Some of the facilitators to condom use were knowledge on HIV, higher perceived HIV risk, peer support, positive manager attitude, health provider engagement, and city ordinances. In contrast, some of the barriers to condom use were discomfort and displeasure on condom use, low parental communication, lack of sex education, social stigma, and the high price of condoms.

**Conclusion**: A collaborative, culturally-sensitive, and population-specific approach is essential to develop and implement acceptable, sustainable, and successful condom use interventions.

## Introduction

In 2018, the Philippines was one of the countries in the world with the highest human immunodeficiency virus (HIV) incidence-prevalence ratio.^1^ Between 2010 and 2018, the Philippines had a 203% increase in new HIV infections.^1^ In August 2019, an average of 36 Filipinos per day was diagnosed with HIV.^2^ In 2018, the country’s proportion of young individuals among total estimated new HIV infections was 69%, the highest in Asia and the Pacific region.^1^ From January to August 2019, HIV was commonly transmitted through sexual contact, mainly among men who have sex with men (MSM) with 5248 reported cases.^2^ Preventive programs and services are significant to be available and accessible for communities and populations-at-risk to alleviate the burgeoning impact of HIV. Some of the strategies to deliver HIV preventive measures are information, education and communication, peer-based approach, and distribution of condoms and other preventive materials.^3^ Abstinence, limiting sexual partners, and use of condoms are some of the specific measures to lessen HIV risk.^4^ The use of condom offers a practical and effective means to prevent HIV transmission.^3^ Based on a systematic review, consistent use of condoms reduces HIV transmission by more than 70% among HIV serodiscordant heterosexual couples.^5^

In the Philippines, the rates of condom use among the populations of injecting drug users (IDUs), transgender people, MSM, and sex workers in 2018 were 14.5%, 37.17%, 49.8%, and 70.6% respectively.^6^ In a large-scale survey participated by 25,074 women aged 15 to 49 years from different regions of the Philippines, only 66% knew that consistent use of condom is a method of preventing the spread of HIV.^7^ Women with older age, higher level of education, higher wealth quintile, and located in urban areas were more likely to know that consistent use of condoms is a method of preventing HIV transmission.^7^ In the early 1990s, the late Department of Health (DOH) Secretary Juan Flavier exerted effort for an aggressive campaign on condom use that focused on sex workers and mobilized former sex workers as educational outreach volunteers even it was opposed by the Roman Catholic Church.^8^ However, social, political, and structural barriers prevent access of Filipinos to effective condom use.^8^ Some of the barriers are restrictive policies in the purchase of condoms in local government units, inadequate sex education in schools, and the resistance of the Roman Catholic Church to condom use.^8^ Factors that facilitate and prevent condom use are necessary to be identified to inform educators, health providers, policymakers, and other stakeholders to determine strategies in creating and implementing programs and services to promote the correct and consistent use of condoms.

### Conceptual framework

The Ecological Model for Health Promotion^9^ was chosen as the framework of the study to identify the factors that influence condom use among Filipinos. It was used to obtain a comprehensive understanding and analysis of different factors at the intrapersonal, interpersonal, institutional, community, and public policy levels that affect condom use.

The model provided a broader perspective of investigating how the interrelationships between the characteristics of Filipinos and the social, cultural, and structural components of their environment can promote, support, or hinder their use of condoms. In this case, gaps in promoting condom use can be further identified and addressed.

The study aimed to contribute knowledge in condom use education and promotion. The purpose of this study was to identify facilitators and barriers to condom use among Filipinos.

## Materials and Methods

### Data collection

**Figure 1:**
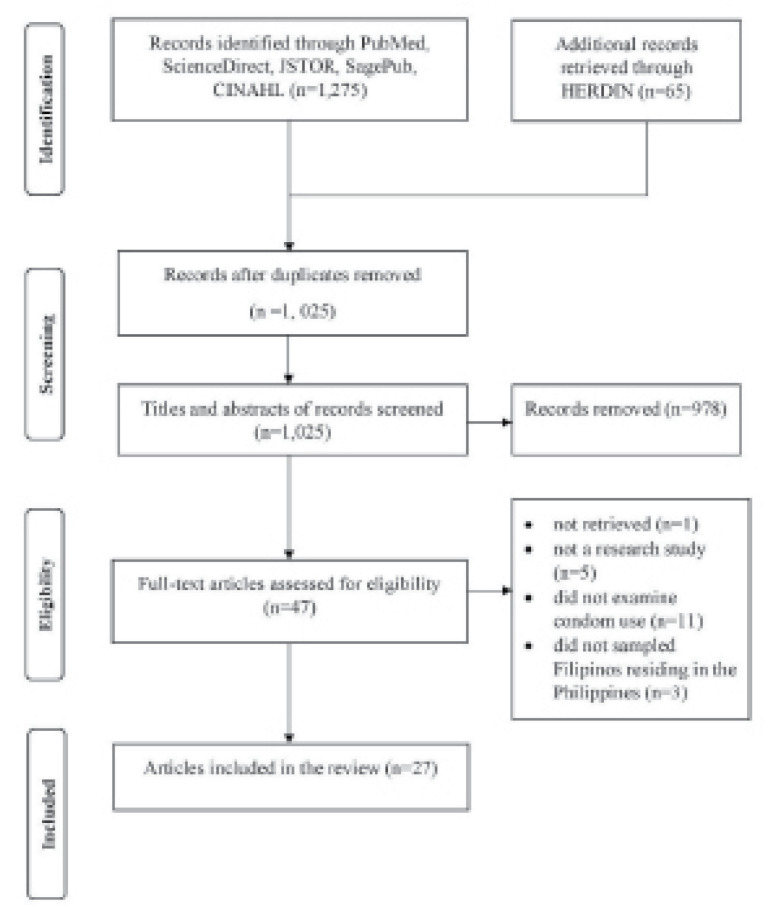
Flow of paper identification, screening, selection, and inclusion

A systematic review of literature was conducted through electronic databases CINAHL, JSTOR, PubMed, SagePub, and ScienceDirect using a combination of keywords, “condom,” “Filipinos,” and “Philippines.” Health Research and Development Information Network (HERDIN), an online portal developed by the Philippine Council for Health Research and Development (PCHRD), was also used to locate other national health studies and related information. The number of records retrieved was 1340, and the number of titles and abstracts initially screened was 1205. To be included in this review, papers should be (1) research studies, (2) studies that examined condom use, and (3) studies that sampled Filipinos residing in the Philippines. A total of 47 papers were examined in full-text for eligibility, and 27 articles were appraised and included in the analysis (Figure 1). Supplementary file 1 shows the complete search strategies used for this review.

### Data analysis

The quality of articles was appraised using the Joanna Briggs Institute’s Critical Appraisal Tools^10-13^ with 8 to 13 items that examine the papers’ validity, methodology, and relevance. Data extraction was done using a table to obtain author and year of publication, study setting, design, sample, methods of condom use assessment, consistency of condom use, and the factors influencing condom use. The articles were read and compared several times. The identified factors were examined for similarities and differences and then grouped based on the assumption of the study framework. Consultation with a public health expert was done to enhance the analysis and interpretation of findings.

## Results

### Sample characteristics

A total of 27 articles were included in this review (Table 1). Most of the studies used quantitative methods. Only two studies used qualitative methods through focus group discussions with heterosexual young adults,^14^ and semi-structured interviews with cisgender-MSM and transgender women.^15^ Most of the studies were conducted in the Southern Philippines, particularly in Cebu. Female sex workers (FSWs) were the most common study samples, while only one study sampled high school students.

### Assessment of condom use 

There are several methods of how condom use was assessed. Study samples were asked using a three to five-point Likert scale or a validated tool to determine the consistency of condom use in the past week, month, or 6 months. Other methods were done by asking the samples whether they had ever used condoms, had used condoms during their last sex, or negotiated condom use within the past 6 months. Variations in the consistency of condom use among the samples were observed.

### Facilitators and barriers to condom use

*Intrapersonal factors*

At the individual level, the factors affecting condom use are sociodemographic characteristics, personal preferences, and perceived sexual pleasure. Other individual factors are HIV and condom use knowledge and attitude, and perceived HIV risk and condom-related stigma. Consistent use of condom was associated with more educational years,^16^ longer employment period,^17^ recent HIV status,^17^ recent HIV status,^17^ frequent sexually transmitted infection (STI) tests,^18^ higher monthly wage,^17^ and being single.^19^ FSWs who were married,^18^ with a regular partner,^20^ and more frequent alcohol drinker^18^ had greater inconsistencies in condom use. To earn additional money was a reason for few FSWs to engage in unprotected vaginal sex occasionally.^21^ Lack of money was a reason for not using condoms for some high school students in a randomized controlled trial study.^22^ In a secondary analysis, male IDUs reported more frequent condom use when having sex with an FSW and with their regular partner.^23^ The lack of condom negotiation among FSWs was associated with the use of an illegal substance.^24^

In several studies, the reasons for not using condoms were did not think of using condoms,^19,25,26^ discomfort and displeasure in condom use,^14,19,26-29^ interruption of sexual activity,^19,26,28^ lack of available condom,^19,25-27^ and use of another contraceptive method.^19,22^ For FSWs,^19^ male sea-based overseas workers^29^ and MSM,^26^ using condoms with sexual partners was a personal decision. In a survey of FSWs on what they intend to do if they were unable to get a condom, the majority answered that they would get a condom in someplace.^21^ Also, most of the women reported that they never had sex without a condom because it was not available.^21^ The use of condoms was a way for young unmarried adults to conceal sexual activity and avoid having a pregnancy and STI.^14^

Among high school students, lack of knowledge on condoms was cited as a reason for not using condoms.^22^ More knowledge on HIV was positively correlated with more positive attitudes on condom use^30^ and more consistent use of condoms.^18,31^ Knowledge of the effectiveness of condoms in HIV prevention was a significant predictor of consistent use of condom.^31-33^ Fear on condom use was observed among young adults with the thought that condoms would break and be left inside the woman during sex.^14^ Condom use was even compared with applying a sock on the penis or eating a banana with a peel.^14^ A positive attitude on condom use was associated with condom use during the last sex.^30,32^ FSWs with more favorable condom use attitudes were more likely to use condoms during sex.^20^

Higher perceived HIV risk was associated with the consistent use of condoms.^16,30^ For a few high school students, one of the reasons for not using condoms was not knowing one’s HIV risk.^22^ For a few MSM, the belief that their sexual partner was HIV negative was a reason for not using condoms.^27^ Less perceived HIV susceptibility was a significant predictor of negative condom attitude.^32^ Perceived stigma on buying and using condoms prevents individuals from using condoms. Some FSWs,^19^ male IDUs,^26^ and young adults^14^ expressed being ashamed of buying condoms. For married young adult couples, husband’s use of condom was perceived as a sign of infidelity.^14^ Women who use condoms were stigmatized as prostitutes, promiscuous, and sexually active.^14^

*Interpersonal factors*

Friends, peers, parental communication, and work coercion are interpersonal factors that influence condom use. In a qualitative study, friends of transgender women and cisgender-MSM were sources of access, information, and motivation to condom use.^15^ Young adults received stories from friends and acquaintances about the presence of holes in condoms.^14^ Peer education helped achieve positive influences on condom use. Peers of FSW groups^30,34,37^ and male groups^35-37^ exposed to an intervention on HIV awareness and education achieved a significant increase in condom use. The intervention involved learning the basics of HIV, establishing an interpersonal relationship, providing support in delivering HIV education, and creating educational materials.^30,34-37^ Among the 682 MSM surveyed in a cross-sectional study, 9.8% agreed that they would not use a condom because most of their peers do not use condoms during sexual intercourse.^25^

Partner’s objection and decision were reasons for not using condoms for some FSWs,^19^ high school students,^22^ and MSM.^25^ Tuason et al^38^ determined a significant association between lack of condom use at last sex and low levels of parental communication among young adults. Among FSWs in Quezon City, job coercion was associated with inconsistent use of condoms^18^ and lack of condom negotiation.^24^

*Institutional factors*

The lack of comprehensive sex education and HIV curriculum was a reason for lack of knowledge on the purpose and proper use of condoms.^15^ Seminars were found to deliver knowledge on condom use mainly for family planning and less as a method for HIV prevention.^15^ The types, practices, and managers of work establishments provide support and encouragement for FSWs to use condoms. Registered FSWs working in casas and massage parlors were more likely to use condoms consistently than women working in bars and nightclubs.^19^ In other studies, FSWs in spa or saunas were more likely to negotiate condom use^24^ and use condoms consistenly^18^ than those women in the night clubs. Consistent use of condoms among FSWs was more evident in establishments with HIV education and condom use policy than those without.^20,21,30,37,39^

Manager’s advice,^33^ favorable manager attitude,^32^ and more frequent manager contact^18^ were significant factors to consistent use of condoms. Managers who received training on HIV education and support to implement workplace HIV education and prevention policies positively influenced the condom use practices of FSWs. Along with this, manager training combined with peer education was seen to be more effective in promoting consistent use of condom among FSWs.^17,30,31,34,37,39^

*Community factors*

Access to merchandise stores and health facilities, social stigma, churches, and community participation are factors within the community that influence condom use. For MSM, the major sources of condoms were drugstores and supermarkets, while few access condoms from private or public health facilities.^25^ For FSWs, social hygiene clinics (SHCs) and drugstores were common sources of condoms.^21^ Ease of accessing condoms in convenience stores was diminished by distant location and discreet placements of condoms.^15^ Engagement with health providers was seen to bring positive outcomes in condom use. A positive association between condom education by health providers and consistent condom use was identified in three studies.^18,31,39^ In a survey of FSWs in a rural setting, health providers were the primary sources of information on condom use.^33^ Health facilities and non-government organizations were reported to provide condom education and free condoms for cisgender-MSM and transgender women.^15^ However, it was found that there were facilities that require HIV testing before receiving free condoms.^15^ Several studies presented the significance of collaboration and participation among academic, health, government and non-government organizations, and work sectors in conceptualizing, developing and implementing interventions to promote condom use among specific populations.^34-37,39^ Support, guidance, and linkages were seen to be instrumental for interventions to achieve positive outcomes toward condom use.^34-37,39^

In a qualitative study, sources of social stigma in accessing and using condoms were health facilities, schools, convenience stores, and churches.^15^ Interrogations of sellers on the reason for buying condoms were stigmatizing experiences reported in two qualitative studies.^14,15^ Condom-related stigma was a reason for lack of discussion on condom use with health providers, and for not getting condoms in health facilities.^15^ Churches in the Philippines prohibited the use of condoms because it was perceived more as a method of contraception than prevention from diseases.^14^ Condom use was reported to be contradictory to the teaching of the Roman Catholic Church on the purpose of sexual engagement for procreation.^14^

*Public policy factors*

The high price of condoms was reported as a barrier to condom use.^15,19,25^ It was described that condoms were expensive, making others not able to afford and use them.^15^ A survey of 1394 FSWs revealed that nearly half reported that the price of condoms was too high to use regularly.^20^ City ordinances existed that required FSWs to be registered in SHCs, acquire a health card, and have a regular visit to SHCs.^18,19,21,24,32,34,39,40^ In Quezon City, an ordinance was enacted to ensure condom availability in registered establishments, mandate HIV/STI education, improve SHC’s examination of female entertainers, implement health policy in work establishments, and prevent the hiring of minor workers.^18,24^ Studies have shown that consistent use of condoms was more evident in registered FSWs than freelance FSWs (see Table 2).^19,40^

## Discussion

Considering the dramatic rise of HIV cases in the Philippines, implementing and strengthening condom use interventions offer valuable means of preventing HIV transmission. However, there are facilitators and barriers to condom use that should be examined and addressed to ensure that interventions and programs achieve notable outcomes. This review supports the principle of the Ecological Model of Health Promotion^9^ on the importance of understanding the interacting factors from the individual and social environment levels that affect condom use. This review presents the multiple interrelated factors that affect condom use among different groups of Filipinos at risk of HIV. From this review, it is learned that experiences, perceptions, and practices of different groups of population on condom use vary not only because of their characteristics, knowledge and attitude, personal decisions and preferences, and perceived HIV risk and condom-related stigma. It is also because of the people they dwell with, the structure, culture, beliefs, practices, and norms of the institution and community they belong, and the governing policies and regulations they need to observe.

Most of the factors that promote and prevent condom use come at the intrapersonal level. Generally, these factors are related to sociodemographic characteristics, personal preferences, and perception of HIV and condoms. These factors could have emanated from other intrapersonal factors. Condom use and HIV awareness campaigns may help individuals realize their HIV risk, clarify their misconceptions on condom use, and encourage them to practice the use of condoms. With inadequate HIV and condom use knowledge, individuals can have misinformation and develop negative attitudes on condom use. Additional intrapersonal circumstances may further hinder the optimal use of condoms. These situations pose a risk for correct and consistent use of condoms. In other cases, the presence of other factors promotes condom use. Among the study samples, registered FSWs had high condom use rates, which can be attributed to their required SHC visits and continuous engagement with health providers. A different approach in providing condom education for MSM might be necessary because of the threat to identity, stigmatization, and discrimination.^41^

At the interpersonal level, friends, peers, and partners promote and support individuals to use condoms. There are also instances that they become the reason for individuals not to use condoms. Friends and acquaintances could be sources of fear, misinformation, and stigma toward condom use that might result in false security and hesitance of using condoms. Couples could interpret the use of condoms as a sign of infidelity rather than a method of preventing HIV and other STIs. This perception limits the choice and drives to use condoms, especially when individuals are in a relationship.

Schools can enable the dissemination of information on the importance and proper use of condoms. However, programs on condom use might not be present, possibly because of the different perceptions of schools to incorporate sexual education. In the Philippines, there are many schools with religious affiliations that can have conservative and varied stands on condom education and distribution. This factor might be a crucial consideration for individuals, especially those students, in learning how they can promote their sexual health and prevent themselves from having HIV. The characteristics of the environment where individuals belong may affect the positive impact of using condoms. Communities’ acceptance and resistance to condom use interventions might vary not only because of their norms and culture but also on the number of their HIV cases. Communities with greater stigma and conservative views may prevent adequate access to condom use interventions. However, if they are educated on the social, economic, and health consequences of HIV, they may become helpful in initiating and advocating condom use promotion and HIV education. The purpose of policies and regulations to promote condom use might not be fully realized if there are communities, groups, or sectors that are resistant to it.

Condom use interventions that focus only on the individual level may not be sufficient to produce transformative and sustainable outcomes. A multisectoral approach of promoting and strengthening widescale campaign and education on condom use showed positive outcomes in terms of consistent use of condoms, reinforcement of condom use policies, and reduction of HIV and STI cases.^42^ A similar strategy has been observed in various studies in this review that utilized a collaborative and community approach by implementing peer education and manager training on HIV awareness and education. Interventions directed at assessing and addressing the different social environment levels could achieve more exceptional and synergistic results.

## Implications ofthestudy

The findings of this review have several implications for education, practice, and research. The assessment of condom use should not only focus on measuring the frequency of condom use. It is suggested to include the assessment of facilitators and barriers to condom use. The use of standardized and multiple questions helps obtain accuracy and consistency in reporting condom use.^43^

A collaborative and culturally-sensitive approach will help educate and engage the multiple sectors of the community to understand their significant roles in condom use promotion and HIV education. This approach can facilitate the effective mobilization of resources to develop innovative, tailor-fit, and sustainable condom use interventions. Dialogue among health providers, government and non-government organizations, educators, religious groups, and other stakeholders can help create comprehensive plans to promote condom use and prevent HIV transmission. This collaboration can also strengthen the reinforcement of existing policies and interventions on condom use and HIV prevention in different populations and communities, especially those at risk.

There is a need to address social stigma to enable individuals to utilize available condom use interventions in their institutions and communities. Investigation of the views and roles of families in condom use promotion and HIV education would be a significant basis on how they could be mobilized as an essential resource of condom use interventions. A trusting environment where individuals can readily access condoms and other HIV preventive measures without the fear of interrogation, stigmatization, and discrimination will be vital to promote correct and consistent use of condoms.

## Conclusion

The Ecological Model of Health Promotion provides a relevant framework to understand and analyze the differences and relationships of individual and social environmental factors that affect condom use. Multiple factors may support and oppose the influence of each other to facilitate or hinder condom use.

Different groups of Filipinos have varying knowledge, attitudes, beliefs, HIV risks, needs, practices, social groups, and environments that influence their condom use. A collaborative, culturally-sensitive, and population-specific approach is essential to develop and implement acceptable, sustainable, and successful condom use interventions.

## Acknowledgment

The author would like to express gratitude to Dr. Bethel Villarta for her valuable support and guidance in writing this manuscript.

## Funding

The author received no financial support.

## Competinginterests

The author declared no competing interest with respect to the research, authorship, and/or publication of this manuscript.

## Ethical approval

Not applicable.

## Author’s contribution

The author conceptualized the study, screened and reviewed the articles, analyzed the data, and wrote and submitted the manuscript.

## Appendix

Appendix 1 contains the complete search strategies for this review.

**Table 1:** Summary of articles

**Author & Year**	**Setting**	**Design**	**Sample**	**Methods of assessment of condom use**	**Consistency of condom use**
Amadora-Nolasco et al (2001)^19^	Cebu	Quantitative: Survey	720 FSWs (360 registered and 360 freelancers)	• Frequency of condom use a week before the interview (always, sometimes, never)	• 46% of registered FSWs always use a condom in all partners • 37% of freelance FSWs always use a condom in all partners
Amadora-Nolasco et al (2002)^28^	Cebu	Quantitative: Survey	360 male IDUs	• Frequency of condom use a month before the interview (always, sometimes, never)	• 5.2% of 250 sexually active male IDUs always use a condom
Amadora-Nolasco et al (2004)^26^	Cebu	Quantitative: Survey	360 MSM	• Frequency of condom use in the past month (always, sometimes, never)	• 11% reported that they always use a condom
Amit et al (2015)^25^	Online dating sites and online MSM communities	Quantitative: Cross-sectional survey	682 MSM	• Condom use at last sex with a male or female partner	• 369 MSM used condom at last anal sex with a male partner • 38 MSM with a female partner used condom at their last sex
Aplasca et al (1995)^22^	Four schools in Metro Manila	Quantitative: Two-arm, randomized, cluster, controlled trial	804 high school students	• Frequency of condom use (always, frequently, seldom, never)	• 9% of 80 sexually active high school students always use a condom
Chiao et al (2009)^17^	Cagayan de Oro, Cebu, Iloilo, and Legaspi	Quantitative: Simple randomized quasi-experimental approach	980 FSWs (baseline assessment)	• Six-item scale on the consistency of condom use	• Postintervention assessment revealed that FSWs in the peer education and manager training group had the highest consistency of condom use mean score (3.04)
Gangcuangco et al (2013)^27^	Metro Manila	Quantitative: Cross-sectional survey	406 MSM	• Consistency of condom use with a male and female partner, and when under the influence of alcohol	• 3% reported consistent use of condom
Guevara et al (2010)^29^	Manila	Quantitative: Cross-sectional survey	100 male sea-based OFWs	• Five-point actual condom use rating scale (never, seldom, sometime, frequent, always)	• The actual condom use rating scores among single and married sea-based OFWs were 2.9 and 3.1 respectively
Liu and So (1996)^40^	Iloilo City	Quantitative: Cross-sectional survey	156 FSWs (110 registered and 46 freelancers)	• Frequency of condom use (always, almost always, sometimes, almost never, never)	• 74% of registered FSWs always or almost always use a condom • 43% of freelance FSWs always or almost always use a condom
Lucea et al (2013)^14^	Metro Cebu	Qualitative	54 young heterosexual adults (26 female and 28 male)	• Focus group discussions on how common young adult couples use a condom, the reason for using a condom, and influencing factors to condom use	• Unspecified
Morisky et al (1998)^20^	Southern Philippines	Quantitative: Survey	1394 FSWs	• Frequency of using condoms in different situations in a five-point scale (always, very often, somewhat often, occasionally, never)	• Of the 825 women who reported if they used condom in their last sex, 69% used condom while 31% did not use a condom
Morisky et al (2004)^35^	Metropolitan Cebu, Legaspi, and Daraga in Bicol Region, Cagayan de Oro City, Cavite City	Quantitative: Comprehensive community-based approach	3389 males (military, police and firemen, industrial workers, taxicab drivers, pedicab drivers, community residents)	• Had ever used a condom • Condom use on last sex	• 21% used a condom the last time they had sex
Morisky et al (2006)^30^	Cagayan de Oro, Cebu, Iloilo, and Legaspi	Quantitative: Quasi-experimental	897 FSWs	• Condom use on last sex with a customer	• Postintervention assessment revealed that FSWs in the combined intervention group had the highest rate (50.8%) of condom use at last sex with a customer
Morisky et al (2005)^34^	Cebu, Iloilo, southern Luzon, and northern Mindanao	Quantitative: Longitudinal study	369 FSWs (baseline assessment)	• Six-item scale on the consistency of condom use	• Postintervention assessment revealed that FSWs in combined peer education and manager training group had the highest condom use assessment score of 27.99
Morisky et al (2010)^31^	Cagayan de Oro, Cebu, Iloilo, and Legaspi	Quantitative: Three-year longitudinal, quasi-experimental study	911 FSWs	• Six-item scale on the consistency of condom use	• Postintervention assessment revealed that FSWs in combined peer education and manager training group had the highest consistency of condom use mean score (3.04)
Morisky et al (2010)^39^	Cagayan de Oro, Cebu, Iloilo, and Legaspi	Quantitative: Community-based participatory research	911 FSWs	• Six-item scale on the consistency of condom use	• Postintervention assessment revealed that FSWs in combined peer education and manager training group had the highest consistency of condom use mean score (3.04)
Morisky et al (2005)^36^	Lapu-Lapu and Mandawe City in Cebu	Quantitative: Quasi-experimental study	700 males (400 taxi drivers and 300 tricycle drivers)	• Five-point scale on frequency of condom use • Condom use on last sex	• Postintervention condom use mean scores of intervention and control group were 2.54 and 2.10 respectively
Morisky et al (2002)^21^	Cebu, Iloilo, southern Luzon, and northern Mindanao	Quantitative: Survey	1394 FSWs	• Frequency of condom use in five-point scale (always, very often, somewhat often, occasionally, never)	• In establishments with condom use rule, 49% of 482 FSWs always use a condom • In establishments without condom use rule, 33% of 287 FSWs always use a condom
Morisky et al (2002)^32^	Social hygiene clinics, residences, and business establishments in the Philippines	Quantitative: Exploratory factor analyses	628 FSWs	• Eight items on condom efficacy and use which ask the frequency of condom use during vaginal sex, frequency in suggesting to use a condom, frequency of carrying a condom, use of a condom at last sex, discuss the use of condom on a customer, frequency of asking a customer to use a condom	• Unspecified
Morisky and Tiglao (2010)^37^	Cagayan de Oro, Cebu, Iloilo, and Legaspi	Quantitative: Quasi-experimental	1284 FSWs and 2436 males	• Six-item scale on the consistency of condom use • Condom use at last sex	• Postintervention assessment revealed that FSWs in the combined intervention group had the highest rate (50.8%) of condom use at last sex with a customer • Postintervention assessment condom usage among male was 38.7%
Nishimura-Takahashi et al (1998)^33^	Tarlac	Quantitative: Cross-sectional survey	121 FSWs	• Frequency of condom use using a five-point Likert scale (always, often, sometimes, seldom, never)	• 38.2% of 110 FSWs who knew what a condom always use a condom during sex with clients
Regan et al (2013)^23^	Albay, Cagayan de Oro City, Cavite City, and Metropolitan Cebu	Quantitative: Secondary analysis of data	2272 males	• Consistency of condom use with FSW and regular partner in a four-point Likert scale (always, very often/almost always, some of the time/occasionally, never)	• 16.17% used a condom in their last sex, 3.18% always use a condom with a regular partner, 45.41% always use a condom with FSWs
Regan and Morisky (2012)^16^	Albay, Cagayan de Oro City, Cavite City, and Metropolitan Cebu	Quantitative: Secondary analysis of data	386 males	• Frequency of condom use during vaginal sex	• 22.3% used a condom every time they had vaginal sex
Restar et al (2020)^15^	Metro Manila	Qualitative: Semi-structured, one-on-one qualitative interviews	23 transgender women and seven cisgender-MSM	• Semi-structured interviews on barriers and facilitators of condom use	• Unspecified
Tuason et al (2017)^38^	the United States and the Philippines	Quantitative: Cross-sectional survey	247 young adults (Philippine-based)	• Consistency of condom use in the past six months • Condom use at last sex	• Unspecified
Urada et al (2012)^24^	Quezon City, Metro Manila	Quantitative: Survey	142 FSWs	• Condom use negotiation with a customer in the past six months	• 76% usually negotiated condom use in the past six months
Urada et al (2013)^18^	Quezon City, Metro Manila	Quantitative: Survey	143 FSWs	• Consistency of condom use in the past six months	• 58% always used condoms while having sex with establishment guests in the past six months

*Note.* FSWs, female sex workers; IDUs, injecting drug users; MSM, men who have sex with men; OFWs, overseas Filipino workers.

**Table 2:** Summary of factors that influence condom use at different socioecological levels

**Socioecological level**	**Facilitators to condom use**	**Barriers to condom use**
Intrapersonal	• being single^19^ • frequent STI tests^18^ • higher educational years^16^ • higher monthly wage^17^ • higher perceived HIV risk^16,30^ • injecting drug users who engage sex with female sex workers and with regular partner^23^ • knowledge of HIV^18,30-33^ • longer employment period^17^ • personal decision^19,21,26,29^ • positive attitude on condom use^20,30,32^ • recent HIV status^17^ • prevention of pregnancy and STIs^14^	• did not think of using condoms^19,25,26^ • discomfort and displeasure on condom use^14,19,25-29^ • fear of condom use^14^ • female sex workers who were married or with regular partner^18,20^ • lack of condom^19,25-27^ • lack of knowledge on HIV and condom use^14,22^ • lack of money^21,22^ • low perceived HIV risk^27,32^ • perceived condom-related stigma^14,19,26^ • use of alcohol^18^ and illegal substance^24^ • use of other contraceptive methods^19,22^
Interpersonal	• peer training and education on HIV^30,34-37^ • peer support^15^	• job coercion^18,24^ • low levels of parental communication^38^ • misinformation on condom use by peers^14^ • preference of partners^19,22,25^ and peers^25^ of not using condoms
Institutional	• manager attitude^32^ and engagement^18,33^ • manager training on HIV education^17,30,31,34,37,39^ • type of work establishment^18,19,24^ • workplace HIV education and condom use rules^20,21,30,37,39^	• lack of sex education and HIV curriculum in school^15^
Community	• access to health facilities and merchandise stores^21,25^ • collaboration among different community sectors^34-37,39^ • health provider engagement^15,18,31,33,39^	• discreet placements of condoms in stores^15^ • distance to stores^15^ • prohibition of the Church on condom use^14,15^ • required HIV testing before receiving condom^15^ • social stigma^14,15^
Public policy	• city ordinances^18,19,21,24,32,34,39,40^ • registrations of sex workers^18,19,21,24,32,34,39,40^	• high price of condoms^15,19,20,25^

**Appendix 1:** Flow of Literature Search and Inclusion

	**Stages of Literature Search**	**Search Terms**	**Filters and Limiters Used**	**No. of Records**
Identification	ELECTRONIC DATABASES:			
	Cumulative Index of Nursing and Allied Health Literature (CINAHL)	condom AND ((Filipino) OR Philippines)	None	36
	JSTOR	condom AND ((Filipinos) OR Philippines)	None	461
	PubMed	((("condom s"[All Fields] OR "condoms"[MeSH Terms]) OR "condoms"[All Fields]) OR "condom"[All Fields]) AND (("filipino"[All Fields] OR "filipinos"[All Fields]) OR (("philippine"[All Fields] OR "philippines"[MeSH Terms]) OR "philippines"[All Fields]))	None	156
	SagePub	[All condom] AND [[All filipinos] OR [All philippines]]	None	613
	ScienceDirect	Title, abstract, keywords: condom AND ((Filipinos) OR Philippines)	None	9
	OTHER SEARCH DATABASES:			
	Health Research and Development Information Network (HERDIN)	condom AND ((Filipino) OR Philippines)	None	65
	TOTAL			1340
Screening	DUPLICATE RECORDS			315
	RECORDS AFTER DUPLICATES REMOVED			1025
	RECORDS REMOVED AFTER INITIAL SCREENING OF TITLES AND ABSTRACTS			978
Eligibility	FULL-TEXT ARTICLES ASSESSED FOR ELIGIBILITY			47
	Not retrieved			1
	Not research studies			5
	Did not examine condom use			11
	Did not sample Filipinos residing in the Philippines			3
Inclusion	TOTAL STUDIES INCLUDED			27
	Quantitative studies included			25
	Qualitative studies included			2
